# Contribution of Filopodia to Cell Migration: A Mechanical Link between Protrusion and Contraction

**DOI:** 10.1155/2010/507821

**Published:** 2010-07-06

**Authors:** Fei Xue, Deanna M. Janzen, David A. Knecht

**Affiliations:** Department of Molecular and Cell Biology, University of Connecticut, Storrs, CT 06269-3125, USA

## Abstract

Numerous F-actin containing structures are involved in regulating protrusion of membrane at the leading edge of motile cells. We have investigated the structure and dynamics of filopodia as they relate to events at the leading edge and the function of the trailing actin networks. We have found that although filopodia contain parallel bundles of actin, they contain a surprisingly nonuniform spatial and temporal distribution of actin binding proteins. Along the length of the actin filaments in a single filopodium, the most distal portion contains primarily T-plastin, while the proximal portion is primarily bound by *α*-actinin and coronin. Some filopodia are stationary, but lateral filopodia move with respect to the leading edge. They appear to form a mechanical link between the actin polymerization network at the front of the cell and the myosin motor activity in the cell body. The direction of lateral filopodial movement is associated with the direction of cell migration. When lateral filopodia initiate from and move toward only one side of a cell, the cell will turn opposite to the direction of filopodial flow. Therefore, this filopodia-myosin II system allows actin polymerization driven protrusion forces and myosin II mediated contractile force to be mechanically coordinated.

## 1. Introduction

Cell migration is a fundamental cellular process essential for embryonic development, wound healing, immune responses, and development of tissues. Almost universally, crawling motility involves a cycle of four steps that spatially and temporally coordinate forces in the actomyosin cytoskeleton with extracellular adhesion: plasma membrane protrusion at the leading edge, formation of new adhesion sites under the protrusion, disruption of older adhesion sites at the cell rear, and contraction resulting in cell body movement [1]. Although many aspects of these processes are understood individually, how they are spatially and temporally coordinated is largely unknown. 

Crawling cells generate two major types of actin-based protrusive organelles, lamellipodia, and filopodia, which have strikingly different actin polymerization machinery and are regulated by different signaling pathways [2–4]. The lamellipodium is characterized by a dense network of short, branched actin filaments, driven by activation of the Arp2/3 complex, followed by filament elongation and barbed-end capping. Addition of actin between the membrane and the ends of the filaments is hypothesized to produce the physical force for protrusion of the membrane at the leading edge [5–7]. 

In contrast, filopodia are transient, thin, hairlike protrusions that contain parallel actin bundles. Filopodia in migrating cells have been proposed to be formed by reorganization of the dentritic network [8]. An alternative model proposes that filopodia are formed through the direct polymerization of parallel actin filament networks by members of the formin family. In many species, Dia, one of the formins, localizes to the tips of filopodia and nucleates parallel actin elongation at the barbed end [9–11]. Lamellipodia frequently also contain parallel actin filament bundles called microspikes that remain embedded in the lamellipodia during continuous protrusion [12]. These microspikes can develop into filopodia when they protrude beyond the leading edge [8]. Bundles of actin filaments called retraction fibers can also be left behind as the lamellipodium retracts. These three types of long, parallel actin bundles found in the lamellipodium are interconvertible organelles [8], therefore, throughout this paper, if not specified otherwise, we will refer to these three types of peripheral actin bundles collectively as “filopodia”.

Some filopodia are oriented perpendicular to the lamellipodium front. These filopodia are stationary with respect to the lamellipodium but not the substrate and their protrusion is exclusively driven by actin polymerization at their tips [13, 14]. Filopodia can be anchored to the substrate by focal complexes and this may restrict their movement [3]. Some filopodia are oblique relative to the leading edge, and move laterally with respect to the substrate and the lamellipodium [14, 15]. This lateral movement of filopodia is characterized by rapid changes in direction and frequent collision and fusion of individual filopodial bundles [15]. 

Cross-linking of actin filaments is proposed to be a critical step in filopodia formation since individual long actin filaments lack the stiffness required for efficiently pushing the membrane [5, 16]. Fascin has been proposed to be the major actin cross-linking protein in filopodia [17–19]. However, many cells types do not express fascin, but do express other actin cross-linking proteins, including *α*-actinin, espin, plastin, and villin. These proteins may be involved in the formation of filopodia especially in cells that do not express fascin [17, 20]. 

In this paper, we seek to understand the molecular mechanisms coordinating filopodial behavior during cell migration. We investigated the behavior and dynamics of filopodia during cell migration using live cell fluorescence microscopy of cells expressing different combinations of fluorescently tagged actin binding proteins, including actin, T-plastin, *α*-actinin 1, coronin 1, and myosin II, paying particular attention to examining changes in filopodial organization or motion that related to cycles of cell migration or cell body translocation.

## 2. Materials and Methods

### 2.1. Materials

All reagents were purchased from Sigma-Aldrich (St. Louis, MO) unless stated otherwise.

### 2.2. Cells

Mouse melanoma cells B16F1 (ATCC CRL-6323) were cultured in Dulbecco's modified Eagle's medium with 10% FBS (Atlanta Biologicals), 2 mM glutamine, 100 *μ*g/mL ampicillin and 100 *μ*g/mL streptomycin at 37°C in the presence of 5% CO_2_.

### 2.3. Fluorescent Protein Constructs and Transfections

The full-length human T-plastin sequence was amplified from the a clone containing PLS3 cDNA (ATCC clone 10437180) by polymerase chain reaction with primers containing the restriction sites *Eco *RI/*Kpn *I and cloned into the pEGFP-C1 vector (Clontech, Palo Alto, CA) to produce pEGFP-T-plastin. This gave a mammalian expression vector which produces GFP-T-plastin under the control of the CMV promoter. An mCherry expression vector, pEF6-mCherry was made by ligating the Bam HI–EcoR1 fragment of pRSET-mCherry [21] containing the mCherry gene into Bam HI and Eco RI digested pEF6-GFP (which removes the GFP gene). The mCherry-T-plastin construct was made by cutting the pEGFP-T-plastin with *Bsr G*I and *Mfe *I and ligating it to the *Bsr G*I/*Eco R*I cut pEF6mCherry vector. This mCherry-T-plastin vector produces mCherry-T-plastin under the control of the EF1*α* promoter. The GFP-actin vector allowing expression from the EF1*α* promoter has been described previously [22]. The mCherry-actin vector was constructed by isolating the Bsr G1-Eco RI fragment of GFP-actin containing the actin coding sequence and ligating it to Bsr G1 and Eco RI cut pEF6-mCherry. The myosin II-GFP, [23], *α*-actinin1-GFP [24]. GFP-vinculin, GFP-paxillin, and GFP-talin [25], zyxin-GFP and coronin 1-GFP [26] probes have been described previously. 

B16F1 cells (50,000) were transiently cotransfected with dual color fluorescent fusion proteins using Lipofectamine or Lipofectamine Plus reagents (Invitrogen, Carlsbad, CA) according to the manufacturer's guidelines. At 12–24 hour after transfection, B16-F1 cells were detached from plastic tissue culture dishes by trypsin-EDTA treatment and plated in CO_2_-Independent medium (Invitrogen, Inc.) containing 10% FBS in Bioptechs Delta T culture dishes (Bioptechs, Inc. Butler, PA). The dishes were precoated with 5 or 10 *μ*g/mL fibronectin (BD Biosciences, Bedford, MA), or 5 or 25 *μ*g/mL laminin (Southern Biotech, Birmingham, Alabama) dissolved in PBS for 1 hour at 37°C. The B16F1 cells were allowed to attach to the surface for 2–6 hours prior to observation. There was no difference in cell behavior or filopodial behavior when cells were plated on fibronectin versus laminin.

### 2.4. Time-Lapse Microscopy

Living cells were observed with a Zeiss Axiovert 200M microscope (Zeiss; Göttingen, Germany) using a 100× oil immersion objective (1.3 NA, Zeiss). The plates were kept at 37°C with a Delta T Open Dish controller and heated lid (Bioptechs Inc., Butler, PA). Since prolonged exposure to intense light led to phototoxicity and bleaching of the observed cells, frames were taken 30 second apart during time lapse recording with minimal light exposure. Image acquisition was done with a Hamamatsu Orca camera controlled by automation routines developed using Openlab software (Improvision, Inc.; Portage, MI). For population movement analysis, B16F1 cells were allowed to adhere for 4 hours to Delta T dishes, that were either untreated or coated with varying concentrations of mouse laminin (Southern Biotech, Birmingham, AL) or human fibronectin (BD Biosciences, Bed ford, MA**)**. Cells were then imaged every 5 min with a 10× objective at 37°C in CO_2_-independent medium in Bioptechs dishes.

### 2.5. Quantification of Cell Movement, Microspikes, and Filopodial Intensities

The cell movement and translocational velocity were measured by manually tracking the displacement of the nucleus of each cell using the Manual Tracking plugin of ImageJ [27]. Velocity versus time graphs, fluorescence intensity graphs were plotted using Excel, and box plots were created using Prism (Graphpad Software, Inc., San Diego, CA). Velocity data was smoothed using a 3 point sliding window average. Microspikes and filopodia were marked with the ROI (Region of Interest) line tool on 8-bit images. Statistical differences between two conditions were determined using Student's *t* test. The statistical analysis was performed and the graphs were created with Excel and Prism Graphpad 4.0 Software.

## 3. Results

### 3.1. Filopodial Behavior and Actin Cross-Linking Protein Localization during Cell Migration Cycle

B16F1 mouse melanoma cells are considered a motile cell type and we have used them as a model for characterizing the dynamics of actin binding proteins during the motility cycle. We first defined the general movement characteristics of this cell line on different surfaces. Long-term time-lapse images were acquired at low magnification for cells plated on untreated glass and various concentrations of laminin or fibronectin (data not shown). Previous studies have shown that cultured vertebrate cells display a biphasic migrational speed response as ECM surface coating concentration is increased, presumably correlated with cell-surface adhesion strength [28–31]. By analyzing images acquired at relatively long time intervals (5 minutes), the analysis measured translocational movement of the cells by tracking the position of the nucleus, rather than measuring centroid shifts associated with shape change and protrusion. The optimal coating concentration was 5 *μ*g/mL laminin or 2.5 *μ*g/mL fibronectin which led to an average B16F1 cell translocation speed of 0.76 ± 0.16 and 0.65 ± 0.11 *μ*m/min, respectively.

**Figure 1:**
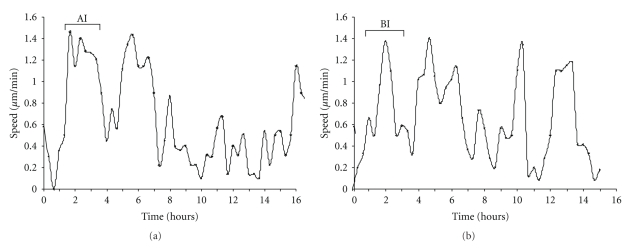
Characterization of B16F1 melanoma cell migration cycles. Variations in speed for a representative cell plated on (a) 5 *μ*g/mL laminin or (b) 2.5 *μ*g/mL fibronectin. Speeds were determined for every 20 min time period over 16 hours of analysis and plotted versus time to show the extent of variation in rate. AI and BI show the time window during the cell migration cycle, respectively that are analyzed in Figure 2.

 The pattern of movement of individual cells under optimal conditions was quite variable. Traces of the speed of a cell measured over 16 hours are shown for representative cells moving on laminin (Figure 1(a)) or fibronectin (Figure 1(b)). The average speed of these cells was within the normal ranges for their respective populations. In both cases, the cells moved in a cyclic pattern with their speeds ranging from approximately 0.1 to 1.5 *μ*m/min. Individual cells could move at a relatively constant rate for periods from 1 to 2 hours, or could show rapid fluctuations in movement. Most importantly, a cell with an average speed at the “slow” end of the distribution was sometimes moving faster than a cell with a high average speed. Given this cyclic migration behavior of cells, it was important to ascertain where in this cycle a cell resides when asking about the relative localization of proteins involved in generating motility. The localization of these proteins may follow a cyclic pattern as well. Thus the analysis of protein localization that follows was correlated with the migration behavior of the cell. 

**Figure 2:**
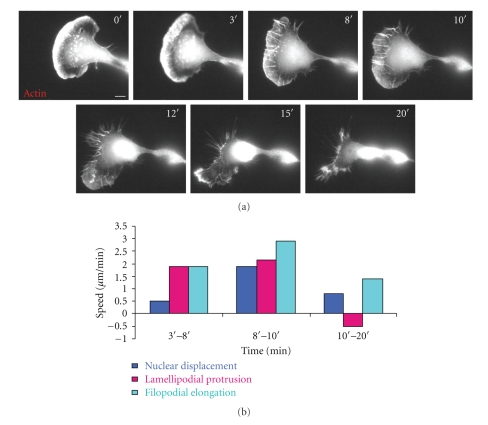
Filopodial behavior during cell migration followed by leading edge retraction. (a) High magnification image sequences of B16F1 cell transiently transfected with mCherry-actin. The projection of filopodia beyond the edge of the lamellipodium followed by retraction of the leading edge. The entire movie can be found in Supplementary Data, Movie 1. (b) Cell retraction, speed and filopodial behavior over three time periods. Light blue, pink and turquoise represent speed of nucleus displacement, lamellipodial protrusion and filopodial elongation, respectively. The frame position is locked so movement toward the edge represents lamellar extension/retraction. Scale Bar 10 *μ*m.

Different cells showed different patterns of internal organization of cytoskeletal protein localization in relation to their movement. We have focused on the organization of the actin cytoskeleton and the filopodia that are sometimes found at the leading edge of the cell. In Figure 2, the localization of mCherry-actin during the motility cycle of a cell that is similar to the B1 time window of the migration cycle in Figure 1(b) is examined. The leading edge of the lamellipodium moved forward from 0 to 10 minutes. Initially, no filopodia were visible, but they began to form at 1.5 minutes, and their number increased from 1.5 min to 10 min (Figure 2(a)). The filopodia began to project beyond the leading edge of the lamellipodium at 8 min. The leading edge then began to retract. During the protrusion phase (from 1 to 8 min), the nuclear displacement speed was low (0.5 *μ*m/min), and filopodia were initiated, elongated and remained within the lamellipodium. The speed of the filopodial protrusion matched that of lamellipodial protrusion (Figure 2 and Supplementary Movie 1 available online at doi: 10.1155/2010/507821).From 8′ to 10′, the filopodia were protruding faster than lamellipodia, and there was a burst of nuclear displacement (1.9 *μ*m/min) (Figure 2 and Supplementary Movie 1). During the retraction phase, while the lamellipodium edge was retracted toward the base of the filopodia, the projected filopodia were still persistently growing. The cell body (nuclear displacement) continued moving forward with a speed 0.8 *μ*m/min. During this time, it seems that there were forces pulling both the cell front and cell rear toward the base of the filopodia resulting in retraction of the front and nuclear displacement toward the base of the filopodia. 

**Figure 3:**
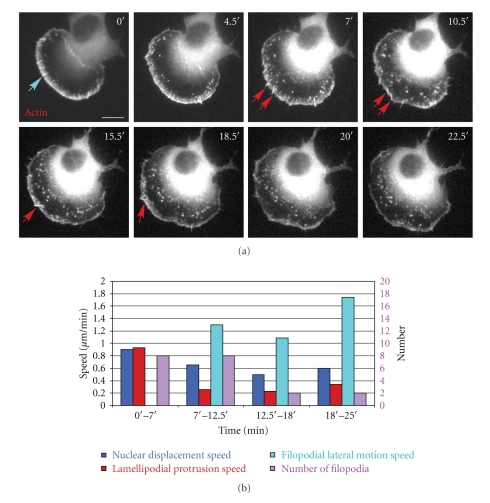
Filopodial behavior during continuous cell movement. (a) The formation and movement of filopodial structures was tracked in GFP-T-plastin/mCherry-actin coexpressing cells migrating at different speeds (0.98, 0.71, 0.56, and 0.66 *μ*m/min during 0′–7′, 7′–12.5′, 12.5′–18′, and 18′–25′, resp.). Only the actin probe is shown. When filopodia started projecting beyond the lamellipodium at 7′, the cell did not start retraction, but instead continued to move forward. Turquoise arrow indicates a stationary filopodium, red arrows represent lateral filopodia. The entire movie can be found in Supplementary Data, Movie 2. (b) Cell protrusion, speed and filopodial behavior. Light blue, red, turquoise and pink column show the speed of nuclear displacement, lamellipodial protrusion, filopodial lateral motion, and the number of stationary and lateral filopodia, respectively. The speed of filopodial lateral motion was measured by the displacement of the distal end of lateral filopodia over time. Scale Bar 10 *μ*m.

Another mode of filoopodial behavior was found in other moving cells. In the cell shown in Figure 3, the number of stationary filopodia (Figure 3(a), turquoise arrow), which were perpendicular to the leading edge and moving or protruding together with the lamellipodium, increased and then decreased while the cell continued to move. During cell movement, the filopodia started projecting beyond the cell membrane at 7.5′. However, instead of retracting like the cell analyzed in Figure 2, filopodia began moving laterally while the lamellipodium continued protruding and the cell continued moving forward (Figure 3, Supplementary Movie 2). Unlike the stationary filopodia, these lateral filopodia moved either along the lamellipodium or toward the cell body and disappeared in the transition zone before reaching the lamella (Figure 3(a), red arrows, and Supplementary Movie 2). The speed of this cell varied; while the number, type, and the speed of filopodial lateral motion were changing as well (Figure 3(b)). In addition, the lateral filopodia changed direction, moving counterclockwise from 7.5 to 10.5 min and then switching to clockwise motion for the duration of the movie. Thus the appearance of filopodia does not necessarily signal the end of the translocation phase of motility. 

**Figure 4:**
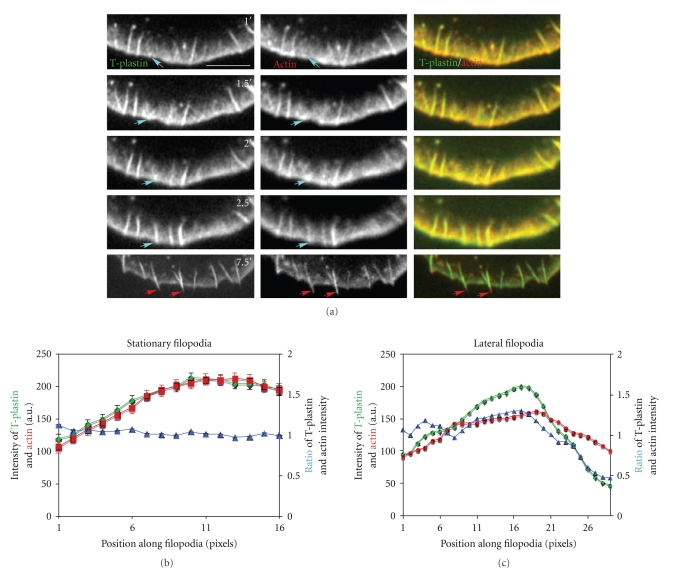
Relative localization of actin and T-plastin to filopodia. (a) Live cell time-lapse of B16F1 mouse melanoma cell co-transfected with GFP-T-plastin and mCherry-actin, showing just the leading edge. Turquoise arrows track a filopodium from initiation of bright dots or short rods, growth of fishtail-shaped filament bundles, and elongatation,. Red arrows indicate lateral filopodia. T-plastin and actin were found throughout stationary filopodia at all stages, but T-plastin was missing from the tips of lateral filopodia. The entire movie can be found in Supplementary Data, Movie 2. (b, c) Quantitative analysis of fluorescence intensity of GFP-T-plastin (green) and mCherry-actin (red) at 1′ for stationary filopodia (b) and 7.5′ for lateral filopodia (c). The *x* axis represents the distance from the proximal to distal end of the filopodia in pixels. Scale Bar 10 *μ*m.

The formation of filopodia could be visualized in cells coexpressing GFP-T-plastin and mCherry-actin. T-plastin is an actin cross-linking protein that strongly localizes to filopodia. Initially, bright dots (Figure 4(a), turquoise arrow, 1′) or fishtail-shaped filament bundles (Figure 4(a), turquoise arrow, 1.5′ and 2′) became visible at the leading edge of the lamellipodium. Subsequently, these structures elongated or fused with each other to form distinct elongated filopodia (Figure 4(a), turquoise arrow, 2.5′). GFP-T-plastin colocalized with the actin probe throughout the entire length of the filopodia, (Figure 4, and Supplementary Movie 2). Analysis of the fluorescence intensities of three randomly chosen stationary filopodia in the 1 min image of the protruding lamellipodium revealed that the pattern of fluorescence intensity of both probes was maximal at the leading edge and gradually decreased toward the proximal end (Figure 4(b)). The ratio of the intensity of the two probes was equivalent along the entire length of the filopodia. However, for the lateral filopodia (Figure 4(a), red arrows, 7.5′), the fluorescence intensity of actin at the tips was much stronger than that of T-plastin (Figure 4(a), 7.5′, red arrow; Figure 4(c)). This came about by the lengthening of the filaments at the distal end of the filopodia with no association of T-plastin with these new filaments, rather than by loss of T-plastin from existing filaments. These results reveal that there may be structural differences in the arrangement of actin filaments in these two types of filopodia that affect the affinity of actin binding proteins. 

**Figure 5:**
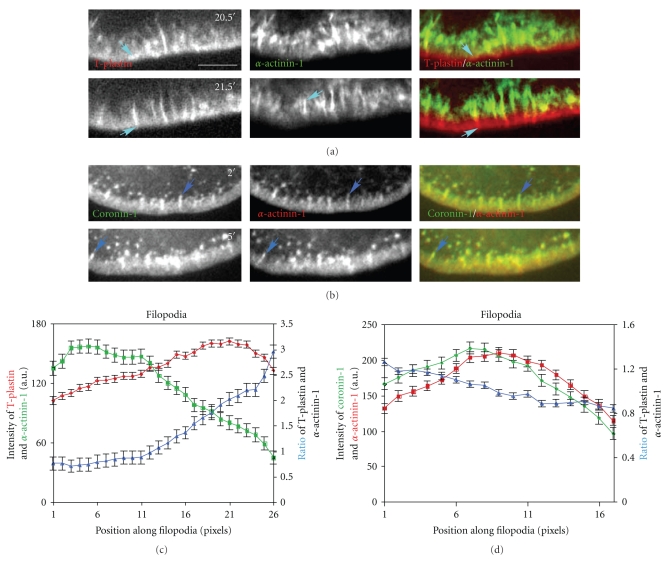
Differential localization of actin cross-linking proteins to lateral filopodia. (a) Live cell time-lapse of B16F1 mouse melanoma cell co-transfected with mCherry-T-plastin and *α*-actinin 1-GFP, showing just the leading edge. Turquoise arrows indicate the elongation of a stationary filopodia. The entire movie can be found in Supplementary Data, Movie 3. (b) Live cell time-lapse of *α*-actinin 1-mCherry and coronin 1-GFP, showing large magnification of leading edge. Light blue arrows are showing elongated filopodia. (c) Average fluorescence intensity of three mCherry-T-plastin and *α*-actinin 1-GFP stationary filopodia, left to right plotting represents proximal to distal of filopodia. (d) Fluorescence intensity of *α*-actinin 1-mCherry and coronin 1-GFP of stationary filopodia, left to right plotting represents proximal to distal of filopodia. Different actin cross-linking proteins preferentially localized to different part of stationary filopodia, T-plastin was stronger at the very distal part, *α*-actinin 1 stronger in the middle part, and coronin 1 stronger at the very proximal part. Scale Bar 10 *μ*m.

We next examined the localization patterns of other actin binding proteins to the filopodia. Coexpression of mCherry-T-plastin and *α*-actinin-1-GFP revealed a differential distribution of these two actin cross-linkers in stationary filopodia during cell migration (Figure 5(a), and Supplementary Movie 3). The *α*-actinin-1 localized strongly to the proximal part of filopodia but was absent from the distal portion. T-plastin was found throughout the entire length of filopodia, but stronger at the distal part where *α*-actinin-1 was absent, and weaker at the proximal portion where *α*-actinin-1 was strongly localized (Figure 5(a)). No localization of *α*-actinin-1 could be seen to the bright dots or short rods or fishtail-shaped filament bundles that initiate filopodial construction. The *α*-actinin-1 probe colocalized with T-plastin from the middle to the base of filopodia, although the fluorescence intensity of *α*-actinin-1 was relatively stronger than that of T-plastin at the base of filopodia (Figures 5(a) and 5(c), and Supplementary Movie 3), indicating a shift in their relative occupancy on actin filaments. In *α*-actinin-1-mCherry and coronin-1-GFP coexpressing cells, GFP-tagged coronin-1 associated with both the lamellipodia and filopodia (Figure 5(b)), but similar to *α*-actinin-1, localized only to the base of the lamellipodia and stationary filopodia. Analysis of three randomly chosen filopodia revealed that the relative fluorescence intensity of coronin-1 and *α*-actinin-1 varied along the length of filopodia. At the base of filopodia, the coronin-1 signal was stronger than that of *α*-actinin-1 but the intensity reversed along more distal portions of filopodia (Figure 5(d)). No obvious differences were observed in the distribution of actin binding proteins along stationary and lateral filopodia. Our results revealed that different actin cross-linking proteins preferentially localize to different parts of filopodia. The most distal portion has primarily T-plastin bound, the middle portion has relatively more *α*-actinin and the proximal portion has all three but a higher relative proportion of coronin-1. Each protein may play a different role in filopodial formation, movement and stabilization. 

### 3.2. Lateral Filopodia Mechanically Link Myosin II with the Lamellipodium

**Figure 6:**
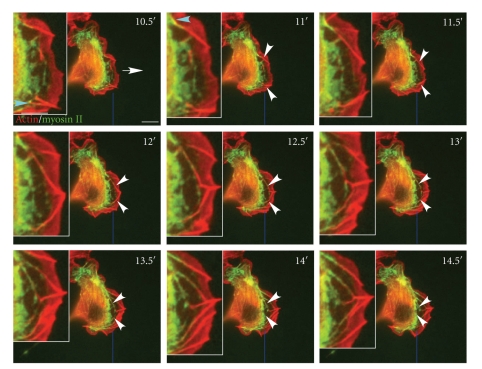
Lateral filopodia and myosin II play important roles in actin-arc formation. (a) Live cell time-lapse of B16F1 cell cotransfected with mCherry-actin and myosin II-GFP. The white arrow shows the direction of cell migration. Turquoise arrowheads show the association of myosin II to filopodia. White arrowheads show the fusion of two converging lateral filopodia. The thick blue line indicates the original position of the leading edge at 0′. The insets show a zoomed in view of the same field. Myosin II associated with the proximal ends of lateral filopodia which subsequently merged and moved into the cell body. The entire movie can be found in Supplementary Data, Movie 4. Scale Bar 10 *μ*m.

We have shown that changes in the cell migration cycle are associated with changes in filopodial behavior (Figures 2 and 3). Different motions of filopodia, such as stationary versus lateral, may represent different forces that a cell generates in order to move more efficiently as it adjusts to its microenvironment. One of the primary force generating molecules that may affect filopod movement is myosin II. We were able to visualize the transformation of lateral filopodia into actin-arcs in B16F1 cells expressing mCherry-actin and myosin II-GFP (Figure 6 and Supplementary Movie 4). This data is consistent with previously published observations on the maturation of these structures [32, 33].

The formation of actin arcs occurred in cycles while the cell moved forward. At the start of a cycle, myosin II was incorporated into a knob-like structure at the base of lateral filopodia (Figure 6, 10.5′ and 11′, turquoise arrowheads). Next, myosin II spread toward the tips of filopodia as they underwent lateral movement (Figure 6(a), 11′–12′). This was sometimes followed by the merger of two or more filopodia (Figure 6, white arrowheads at 12′–13′). Finally, merged filopodia coated with bound myosin II formed the completed actin arcs (Figure 6, 13.5′–14.5′). These actin arcs ended up in the cell body by a combination of rearward transport and forward movement of the cell (Figure 6, 13.5′–14.5′, and Supplementary Movie 4). As the cell moved forward, new lateral filopodia were formed on both sides of the cell and continued merging and moving rearward. Thus myosin II does not appear to be necessary for lateral movement of filopodia, but appears to be involved in the transition of filopodia into actin arcs in the cell body. In order to explore the role of myosin II in the formation of actin arcs, cells expressing mCherry-actin and myosin II-GFP were treated with 50 *μ*M blebbistatin. Before treatment, the filopodial lateral motion and the association of myosin II with the filopodia could be observed. After 10′ treatment, neither new nor preexisting actin arcs could be observed (data not shown). Since actin arcs rapidly disappear in the presence of blebbistatin there was no way to determine whether inhibiting myosin II affected their movement.

 In Figure 3(a), the lateral filopodia did not become actin arcs whereas in the cell in Figure 6 they did. To clarify which lateral filopodia could become actin arcs, we examined many cells that coexpressed myosin II-GFP and mCherry-actin or myosin II-GFP and mCherry-T-plastin. In the cells with large fan-shaped lamellipodia with a large transition zone, the myosin II did not reach and could not associate with the proximal end of the lateral filopodia. These lateral filopodia, which do not associated with myosin II, do not become actin arcs, but rather disappear in the transition zone (Supplementary Movie 5). However, in cells with irregularly shaped lamellipodia, relatively small lamellae and narrow transition zones, the myosin II reached and associated with the proximal end of the lateral filopodia. These filopodia, which associated with myosin II, frequently become actin arcs (Supplementary Movie 6). 

**Figure 7:**
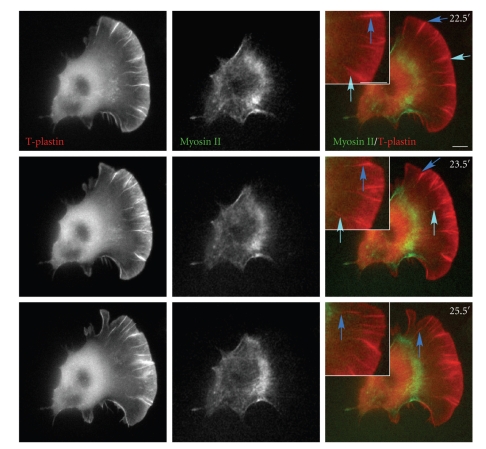
Stationary filopodia are not associated with myosin II. Live cell time-lapse images of a cell cotransfected with myosin II-GFP and mCherry-T-plastin. Arrows show two different thickened filopodia before or after being pulled out of the lamellipodial network. Most stationary filopodia disappeared in the transition zone and could not be tracked moving into the lamella. None of the filopodia showed obvious association with myosin II-GFP. The entire movie can be found in Supplementary Data, Movie 7. Scale Bar 10 *μ*m.

In order to address the question of whether myosin II could associate with stationary filopodia, we examined the localization of myosin II to stationary filopodia in cells with large fan-shaped lamellipodia and broad transition zones or irregular shaped lamellipodia and narrow transition zones. In cells with fan-shaped lamellipodia and broad transition zones (Figure 7), T-plastin localized strongly to the leading edge, whereas myosin II was present at the back of the cell. The cell protruded persistently from the beginning of this image series, and then began retraction at 25.5′ (Figure 7, and Supplementary Movie 7). During lamellipodial protrusion, most stationary filopodia were initiated, elongated and moved forward together with the lamellipodium. However, a few of the stationary filopodia were observed to thicken and then be pulled out of the lamellipodial actin network and move into the transition zone (Figure 7, 23.5′ and 25.5′, arrows). No association of myosin II to the filopodia could be observed regardless of whether these filopodia were rearward moving or not. In comparison, in cells with irregularly shaped lamellipodia and very narrow transition zones, myosin II appeared associated with the stationary filopodia (Supplementary Movie 8). Thus the association of myosin II with filopodia seems to be dependent on the shape of the lamellipodium and the broadness of the transition zone, not on the motion of filopodia. Both myosin II and filopodia are important for the formation of actin arcs, however, not all lateral filopodia or myosin II associated filopodia became actin arcs. Only myosin II associated lateral filopodia were ever observed to become actin arcs. 

**Figure 8:**
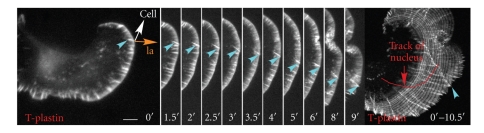
The lateral motion of filopodia is related to the direction of cell migration. Live cell time-lapse of a cell transfected with mCherry-T-plastin. Turquoise arrowheads indicate the lateral filopodia. The golden arrow shows the direction of movement of the original lamellipodium and the white arrow shows the direction of eventual cell migration. The last panel shows a Z-projection of all the images to visualize the track of the nucleus (red line) and the track of the lateral filopodium. The lateral filopodium moves in a direction opposite to the direction of cell turning. The entire movie can be found in Supplementary Data, Movie 9. Scale Bar 10 *μ*m.

In Figure 6, there were lateral filopodia initiated from both sides of the cell moving laterally along the lamellipodium and then merging and moving into the cell body while the cell continued to move forward. We next studied the motion of filopodia in cells which were turning. These cells had laterally moving filopodia, but in this case, instead of merging and converging, the filopodia were all moving in the direction opposite to the turn. In a mCherry-T-plastin expressing cell (Figure 8 and Supplementary Movie 9), the laterally moving filopodium was initiated at the top of the cell (Figure 8, turquoise arrowhead), and moved clockwise along the lamellipodium (Figure 8, turquoise arrowhead). The cell was neither moving in the direction of the original lamellipodial protrusion (Figure 8, golden arrow) nor in the direction of lateral moving filopodia (Figure 8). Instead, the cell (Figure 8, white arrow) was turning counterclockwise and moving forward while the lateral filopodia was moving clockwise. There was no association of myosin II with these laterally moving filopodia, indicating that this type of movement was associated with some other force generating system (Supplementary Movie 10).

**Figure 9:**
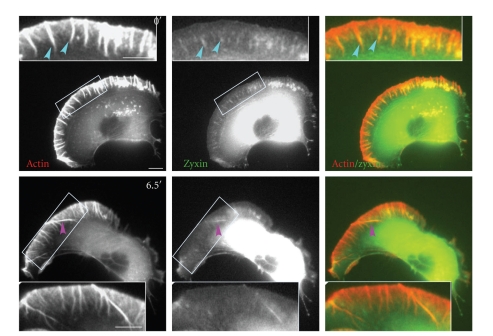
Lateral filopodia and adhesion proteins. Live cell time-lapse of a cell co-transfected with mCherry-actin and zyxin-GFP. Turquoise arrowheads indicate the localization of zyxin to the proximal end of stationary filopodia. Pink arrowheads indicate a lateral filopodium which is associated with zyxin along its entire length. White boxes indicate the areas that are enlarged in order to show the details of stationary and lateral filopodia. The entire movie can be found in Supplementary Data, Movie 11. Scale Bar 10 *μ*m.

Activation of cdc42 leads to the assembly of vinculin containing focal complexes at the cell periphery and along and at the tips of growing filopodia [3]. It was suggested that these areas of close contact might provide transient anchorage sites for forward protrusion of filopodia during migration [34]. We have examined whether molecular markers of adhesions are associated with lateral filopodia using GFP-tagged adhesion protein markers, including vinculin [35], talin [36] paxillin [37, 38], and zyxin [39]. No obvious localization of talin, paxillin, or vinculin to lateral filopodia could be observed, although they all localized to stationary filopodia (data not shown). Zyxin localized to both stationary and lateral filopodia. In the mCherry-actin and zyxin-GFP coexpressing cells, zyxin colocalized with actin at the proximal part of stationary filopodia (Figure 9, 0′, turquoise arrowheads and Supplementary Movie 11) and transiently along the length of lateral filopodia (Figure 9, 6.5′, pink arrowheads). However, the presence of zyxin did not represent adherence to the substratum since zyxin-associated filopodia continued to move laterally.

## 4. Discussion

Filopodia seem to be used by many cell types as a sensing organelle to explore the extracellular matrix (ECM) and the surface of other cells, identify appropriate targets for adhesion, and then generate guidance cues and traction forces to move the cell body [40, 41]. In recent years, much work has been focused on the mechanisms of initiation and formation of filopodia, yet how they function once formed is still largely unknown. It is clear that a number of distinct structures can be classed together under the name of filopodia. The molecular origins of each of these structures is not well understood. Some may arise from formin mediated nucleation, while others may occur from cross-linking of existing actin filaments to form bundles. 

Our results support a biomechanical view of the coordination of the leading edge protrusion and myosin II contractility of migrating cells. In this view, filopodia form a mechanical link between the actin polymerization network at the leading edge and the myosin motor activity at the back. As filopodia originate from the actin dendritic network within lamellipodia [8], they are presumed to be directly connected to the lamellipodial actin network. Filopodia are also the initiation sites for adhesions. Therefore, when a motor protein binds to filopodia and generates contractile forces, the forces will be passed on to adhesions and lamellipodia. Therefore, this filopodia-myosin II system allows actin polymerization, myosin II force, and adhesion to be mechanically coordinated. 

Filament bundling is required for filopodial stabilization, as long actin filaments are not efficient at pushing. It has been shown that fascin is the major actin cross-linking protein in filopodia [17, 18] and is essential for filopodia formation [19]. Our results show that T-plastin may play an important role in filopodia formation as well. It is present throughout the lifetime of the filopodia, from the fish-tail-shaped initiation points, through elongation and movement. As the convergence model specified, the cone-shaped or fish-tail-shaped structures are a prerequisite for filopodia initiation [8]. To allow for efficient pushing, cross-linking of growing filaments is predicted to occur soon after polymerization so that the effective length of individual filaments after the last cross-link remains short. The fact that T-plastin is enriched in the distal section of filopodia and lamellipodia suggests that the association of T-plastin with the growing actin bundles occurs in parallel with actin assembly. T-plastin is likely to be one of the components of the filopodial tip complex responsible for linking the barbed ends of actin filaments. T-plastin is present in both lateral and stationary filopodia, implying it is important in filopodia formation and movement. 

Other actin binding proteins also associate with filopodia, but there is an inhomogeneous distribution of actin binding protein along the length of the filopodia. T-plastin is generally associated with the entire filament bundle, except at the tips of lateral filopodia, while *α*-actinin is found in the middle section, and coronin 1 more at the base. T-plastin and *α*-actinin belong to the same calponin-homology (CH) domain superfamily with a highly conserved F-actin binding domain (ABD) [42–47]). Coronins are members of a highly conserved family with a conserved basic N-terminal motif and three to ten WD repeats clustered in one or two core domains (Uetrecht AC & Bear JE, 2006). They bind filamentous actin and the Arp2/3 complex and play an important role in lamellipodial protrusion and whole-cell motility [48–50]. Since filopodia are presumed to contain continuous long actin filaments, the mechanism by which these proteins differentially bind the different parts of the same actin filaments is of great interest. The binding properties of the ABP's may be regulated in some way within the filopodium, or there may be some difference in the actin filament itself that alters its affinity for different binding proteins. It is noteworthy that binding of proteins to actin filaments can change the twist of the actin helix and alter the binding affinities of other molecules [51, 52]. It is also possible that the ATP/ADP state of the nucleotide bound to actin is important for regulating protein binding to filaments [53]. 

Our results have shown that filopodia can be persistent and escape depolymerization after associating with myosin II and moving into the cell body to form actin arcs. These results are in agreement with Nemethova et al. [33] but differ from those of Medeiros et al. who found myosin II severing actin bundles at the base of lamellipodia [54]. During the cell migration cycle, stationary filopodia are initiated and elongated while the cell continues to move forward. Next, the stationary filopodia begin to project beyond the leading edge. At this point, the cell will either continue to protrude and migrate with filopodia moving laterally, or start retracting the leading edge. While the leading edge is retracting, the cell body could still translocate forward until the retraction stops. This indicates that without actin polymerization, retraction alone can result in cell body translocation. Thus, forces generated by leading edge protrusion or cell body contraction can lead to forward movement. 

Previously, myosin II has been reported to be absent from lamellipodia of fish keratocytes [55]. However, actin and myosin II displayed a highly correlated distribution in the transition zone between the lamellipodium and the cell body in rat embryo fibroblasts [56], fish epidermal keratocytes [55], and neuronal growth cones [54], where the two proteins concentrate in distinct arc-shaped fibers [54–56]. Arc-shaped bundles of actin filaments can be frequently observed beneath the dorsal surface of the lamella of spreading and migrating cells [57, 58]. These actin arcs are parallel to the leading lamellipodia [58]. Hotulainen and Lappalainen reported that actin arcs are generated from *α*-actinin-decorated actin filaments and assemble endwise with myosin bundles to form contractile structures [59]. However stress fibers are unlikely to play a significant role in highly motile cells. The finding that myosin II and lateral filopodia participate in actin-arc assembly was shown by [15, 32, 33]. Here we provided more details about the association of myosin II with newly formed arcs. We hypothesize that lateral filopodia, which link myosin II and lamellipodia, can generate a biomechanical force at the leading edge of a cell. To support this hypothesis, we studied the movement and shape of many cells with lateral and/or stationary filopodia and their relationship with myosin II. When cells have a large, fan-shaped lamellipodium and a broad transition zone, myosin II's localization seems to be limited to the cell body and lamella, and is not associated with either lateral or stationary filopodia. When cells have small, irregularly shaped lamellipodia and a narrow transition zone, myosin II localizes not only to the cell body and lamella, but also the back of the lamellipodium. Myosin II in these cells can associate with filopodia, both lateral and stationary. Therefore, the association of myosin II with filopodia in a cell is dependent upon the shape of the lamellipodium, and the broadness of the transition zone. However, the formation of actin arcs is dependent upon the lateral movement of filopodia and the association of myosin II with them. The lateral filopodia-myosin II system could provide additional forces for these cells that are without perfect lamellipodia moving forward. Actin cross-linking proteins can bundle long actin filaments together to form filopodia, which not only become efficient at pushing the cell membrane, but also efficient at pulling the cell body. In our filopodia-myosin II model, myosin II is linked to filopodia in the lamellipodium and the actin cortex and adhesions in the lamella and cell body. This would allow myosin II to provide the actual pulling forces to retract the cell body. 

Filopodia were found to have three modes of behavior in these cells. In some cells, they elongated and then disappeared without moving. When laterally moving filopodia appeared, in some cases they moved from both sides and converged at the center of the cell, and in other cases all filopodia move in one direction. If there was no filopodial lateral motion or there was converging motion, the cell usually would continue moving straight forward. The filopodial movement could either be a consequence of forces applied to the cytoskeleton, or part of the force generating mechanism. We hypothesize that when lateral filopodia are initiated from both sides of a cell, the forces generated by the lateral motion of filopodia are balanced, thus the cell can move straight forward. When the lateral filopodia are initiated from one side of a cell, the forces applied on the cell are unbalanced and the cell turns. We propose a model in which the distal ends of lateral filopodia are associated with the actin meshwork of the lamellipodium and the proximal ends are associated with the cellular cytoskeleton. When the lateral filopodia are initiated from one side of a lamellipodium, the distal ends are moving along the lamellipodium and result in a force applied to the lamellipodium through the proximal end. The lamellipodial protrusion continues but the protrusive force of actin polymerization is now affected by or combined with the force from the lateral filopodia, consequently the cell changes shape and turns. Thus, cells do not simply move in the direction of actin polymerization or the direction of contractile force, but rather they move in the direction that is the sum of the various forces. Thus, it is likely that the coordination of actin polymerization, adhesion dynamics and myosin activity modulate cell migration velocity and direction.

## Supplementary Material

Supplementary Movie 1: B16F1 mouse melanoma cell expressing mCherry-actin undergoing random migration on 5 *µ*g/ml laminin coated glass surface. Images were taken at 30 s interval on a Zeiss Axiovert 200M microscope using a 100× oil immersion objective.
Supplementary Movie 2: B16F1 mouse melanoma cell expressing GFP-T-plastin and mCherry-actin migrating on 5 *µ*g/ml laminin coated glass surface. Left panel: GFP-Tplastin, Middle panel: mCherry-actin, Right Panel: color merge. This movie shows the changes in the type and number of filopodia during cell migration. Images were taken at 30 s intervals on a Zeiss Axiovert 200M microscope using a 100× oil immersion objective.
Supplementary Movie 3: B16F1 mouse melanoma cell expressing mCherry-T-plastin (left panel) and GFP-*α*-actinin-1 (middle panel) migrating on 5 *µ*g/ml laminin coated glass surface. T-plastin and *α*-actinin-1 both localize to filopodia and the lamellepodium, but T-plastin was stronger at the distal edge, while *α*-actinin-1 was stronger at the proximal region of the lamellipodium and filopodia. Images were taken at 30 s intervals on a Zeiss Axiovert 200M microscope using a 100× oil immersion objective.
Supplementary Movie 4: B16F1 mouse melanoma cell expressing mCherry-actin and GFP-myosin II migrating on 5 *µ*g/ml laminin coated glass surface. RGB merged images of the two channels are shown. Lateral filopodia in small, irregularly shaped lamellipodia with a narrow transition zone became associated with myosin II and then formed actin arcs. Images were taken at 30 s intervals on a Zeiss Axiovert 200M microscope using a 100× oil immersion objective.
Supplementary Movie 5: B16F1 mouse melanoma cell expressing mCherry-T-plastin and GFP-myosin II migrating on 5 *µ*g/ml laminin coated glass surface. Lateral filopodia are found in the large, fan-shaped lamellipodium. Myosin II was not associated with filopodia. RGB merged images were taken at 30 s interval on a Zeiss Axiovert 200M microscope using a 100× oil immersion objective.
Supplementary Movie 6: B16F1 mouse melanoma cell expressing mCherry-actin and GFP-myosin II migrating on 5 *µ*g/ml laminin coated glass surface. This movie shows lateral filopodia in small, irregularly shaped lamellipodia with a narrow transition zone in which myosin II became associated with filopodia. Images were taken at 30 s intervals on a Zeiss Axiovert 200M microscope using a 100× oil immersion objective.
Supplementary Movie 7: B16F1 mouse melanoma cell expressing mCherry-T-plastin (left panel) and GFP-myosin II (middle panel) migrating on 5 *µ*g/ml laminin coated glass surface. Myosin was found in the rear and not associated with stationary filopodia in the large, fan-shaped lamellipodium or in the broad transition zone. RGB merged images were taken at 30 s intervals on a Zeiss Axiovert 200M microscope using a 100× oil immersion objective.
Supplementary Movie 8: B16F1 mouse melanoma cell expressing mCherry-actin and GFP-myosin II on 5 *µ*g/ml laminin coated glass surface. Stationary filopodia are found in small, irregularly shaped lamellipodia and a narrow transition zone in which myosin II was associated with filopodia. RGB merged images were taken at 30 s intervals on a Zeiss Axiovert 200M microscope using a 100× oil immersion objective.
Supplementary Movie 9: B16F1 mouse melanoma cell expressing mCherry-T-plastin migrating on 5 *µ*g/ml laminin coated glass surface. The cell turns counterclockwise while the laterally moving filopodia are moving clockwise. Images were taken at 30 s intervals on a Zeiss Axiovert 200M microscope using a 100× oil immersion objective.
Supplementary Movie 10: B16F1 mouse melanoma cell expressing mCherry-*α*-actinin-1 and GFP-myosin II migrating on 5 *µ*g/ml laminin coated glass surface. This movie shows a cell turning in the direction opposite of the laterally moving filopodia. There is no association of myosin II with these filopodia. RGB merged images were taken at 30 s intervals on a Zeiss Axiovert 200M microscope using a 100× oil immersion objective.
Supplementary Movie 11: B16F1 mouse melanoma cell expressing mCherry-actin and GFP-zyxin migrating on 5 *µ*g/ml laminin coated glass surface. Left panel: mCherry-actin, middle panel: GFP-zyxin, right panel: RGB merge. Zyxin is localized to the proximal part of stationary and lateral filopodia. Images were taken at 30 s intervals on a Zeiss Axiovert 200M microscope using a 100× oil immersion objective.
